# El Coste de la Calidad de Vida en la Enfermedad de Parkinson Avanzada: Estrategias Eficientes Para Abordar la Enfermedad

**DOI:** 10.31083/RN33482

**Published:** 2025-07-23

**Authors:** Nuria García-Agua Soler, Lucía García Trujillo, Antonio J García-Ruiz

**Affiliations:** ^1^Economía de la Salud y Uso Racional de Medicamentos, Departamento de Farmacología, Facultad de Medicina, 29010 Málaga, España; ^2^Instituto de Investigación Biomédica de Málaga (IBIMA), Universidad de Málaga, 29016 Málaga, España; ^3^Servicio de Neurología, Hospital Regional Universitario de Málaga, 29010 Málaga, España

**Keywords:** evaluación económica, enfermedad de Parkinson, años de vida ajustados por calidad, cost-benefit analysis, Parkinson disease, quality-adjusted life years

## Abstract

**Introducción:**

A medida que la enfermedad de Parkinson avanza, se desarrollan síntomas que hacen más difícil el control de la misma, bien por la presencia de fluctuaciones a pesar de un tratamiento adecuado oral, bien por los efectos adversos provocados por el uso continuado de levodopa, considerado el Gold standard. Llegado ese momento en el curso de la enfermedad, existen diferentes alternativas terapéuticas que proporcionan una estimulación dopaminérgica más continua y ayudan a mejorar esta sintomatología: estimulación cerebral profunda (Medtronic®: Percept™ Primary Cell (PC), Percept™ ReChargable (RC)), gel intestinal Levodopa/Carbidopa (Duodopa®) o Levodopa/Carbidopa/Entacapona (Lecigon®), así como la infusión subcutánea de foslevodopa/foscarbidopa (Foslevodopa®) y la infusión continua de apomorfina (Dacepton®, Apo-Go®).

**Objetivo:**

Estudio farmacoeconómico de las diferentes terapias para tratar la enfermedad de Parkinson avanzada en España.

**Pacientes y Métodos:**

A partir de un modelo de Markov, se compararon las eficacias y los costes de estas terapias, midiendo años de vida ganados (AVG) y años de vida ajustados por calidad (AVAC).

**Resultados:**

Dacepton® es la opción más coste-efectiva con un coste de 20.782€/AVAC (1€ = 1,0815 USD, 2025), frente a tres veces o más del resto de terapias siendo Lecigon® la menos rentable con un coste de 104.000€/AVAC. La deep brain stimulation-estimulación cerebral profunda (DBS) y el Duodopa® también demostraron ser opciones eficaces, pero más costosas que Dacepton®.

**Conclusiones:**

Estos resultados permiten obtener información adicional sobre la eficiencia de los tratamientos que deben servir en la toma de decisiones en el manejo de la enfermedad de Parkinson avanzada, y de este modo conseguir una mejor gestión de los recursos.

## Abstract (en)

**Introduction:**

As Parkinson’s disease progresses, symptoms develop that make it more difficult to control, either because of the presence of fluctuations despite adequate oral treatment, or because of the adverse effects caused by the continued use of levodopa, considered the gold standard. At this point in the course of the disease, there are different therapeutic alternatives that provide more continuous dopaminergic stimulation and help to improve this symptomatology: deep brain stimulation (Medtronic®: Percept™ Primary Cell (PC), Percept™ ReChargable (RC)), intestinal gel Levodopa/Carbidopa (Duodopa®) o Levodopa/Carbidopa/Entacapona (Lecigon®), as well as subcutaneous infusion of foslevodopa/foscarbidopa (Foslevodopa®) and continuous infusion of apomorphine (Dacepton®, Apo-Go®).

**Objective:**

Pharmacoeconomic study of the different therapies for treating advanced Parkinson’s disease in Spain.

**Patients and Methods:**

Based on a Markov model, the efficacy and costs of these therapies were compared, measuring life years gained (LYG) and quality-adjusted life years (QALY).

**Results:**

Dacepton® (apomorphine) is the most cost-effective option with a cost of 20,782€/QALY (1€ = 1.0815 USD, 2025), compared to three times or more for the other therapies with Lecigon® (levodopa/carbidopa/entacapone) being the least cost-effective with a cost of 104,000€/QALY. Deep brain stimulation (DBS) and Duodopa® also proved to be effective options, but more costly than Dacepton®.

**Conclusions:**

These results provide additional information on the efficiency of treatments that should inform decision-making in the management of advanced Parkinson’s disease, thus enabling better resource management.

## 1. Introducción

La mayoría de pacientes con enfermedad de Parkinson (EP) tratados con levodopa responde satisfactoriamente, pero a medida que va avanzando van apareciendo otros síntomas, además de presentar fluctuaciones de la respuesta motora, como discinesias y distonía, y no motoras y de expresividad neuropsiquiátrica, deterioro cognitivo, psicosis o demencia, trastornos del estado de ánimo y problemas de sueño [1, 2, 3, 4].

En la práctica clínica, cuando las fluctuaciones motoras y no motoras no pueden mejorarse con terapia oral/transdérmica optimizada, los pacientes se clasifican como pacientes con EP avanzada [5, 6, 7]. Para estos pacientes en los que existe incapacidad de controlar adecuadamente los síntomas de la EP avanzada, la terapia asistida por dispositivos puede considerarse una opción de tratamiento alternativo, entre ellos se incluyen: la estimulación cerebral profunda (Medtronic®: Percept™ Primary Cell (PC), Percept™ ReChargable (RC)), gel intestinal Levodopa/Carbidopa (Duodopa®) o Levodopa/Carbidopa/Entacapona (Lecigon®), así como la infusión subcutánea de foslevodopa/foscarbidopa (Foslevodopa®) y la infusión continua de apomorfina (Dacepton®, Apo-Go®).

Aunque estas terapias asistidas por dispositivos son eficaces, los pacientes no siempre están dispuestos a considerarlas, ya sea por su carácter invasivo (miedo a la cirugía y/o complicaciones asociadas, preocupaciones estéticas) o por sus posibles efectos secundarios. También hay que considerar que la disponibilidad está relegada a centros especializados en algunas áreas sanitarias y que la aplicación regional de directrices internacionales sobre el uso de terapias avanzadas en la EP impactan aún más en la consideración de estas terapias, tanto en pacientes como médicos [8, 9, 10, 11].

El objetivo de este trabajo es realizar una evaluación económica de las alternativas disponibles para el tratamiento de la EP avanzada.

## 2. Sujetos y Métodos

Con la misma metodología seguida en un trabajo anterior [12] se procedió a realizar una evaluación farmacoeconómica con la siguiente información:

– Medidas de resultado: datos de eficacia (AVG – años de vida ganados) y de utilidad (AVAC – años de vida ganados ajustados por calidad) medida con la escala de Hoehn y Yahr (H&Y) [13, 14], utilizando el método estándar en los estudios de farmacoeconomía [15, 16]. Toda la información se obtuvo de un trabajo previo publicado en 2016 [12].

– Datos de recursos sanitarios empleados y costes de las alternativas comparadas. Basándonos en el estudio sobre costes de EP publicado en España (estudio eStudio COstes Parkinson Enfermedad (SCOPE) [17]) se valoraron el uso de los recursos asociados a la aplicación de cada uno de los tratamientos y se calcularon los costes totales para el período de pretratamiento, procedimiento y seguimiento del paciente durante 5 años (horizonte temporal). Todos los costes se actualizaron a diciembre de 2023 a partir de los datos ofrecidos por el Instituto Nacional de Estadística (España) [18] y tomados de fuentes directas de costes públicas del Sistema Sanitario Público Andaluz [19, 20] (Tabla 1, Ref. [19, 20]).

**Tabla 1.** **Recursos sanitarios y costes empleados en el tratamiento de la EP avanzada durante 5 años (en € 2023)**.

Procedimiento clínico	Coste unitario (€) [19, 20]	Apomorfina		Medtronic®		LCIG	FosLD/FosCD SC	LCEIG
		Apo-Go® perfusión	Dacepton® perfusión	Percept™ PC	Percept™ RC	Duodopa® gel intestinal	Foslevodopa®	Lecigon®
1ª consulta neurología	117,81	1	1	1	1	1	1	1
Revisión neurología	58,90	9	9	7	7	7	7	7
1ª consulta neurocirugía	153,86			1	1			
Revisión neurocirugía	73,93			1	1			
Valoración psicología	53,51			1	1			
Ingreso neurología	466,5					1		1
Test apomorfina	147,66	1	1					
Ingreso neurocirugía	635,65			7	7			
PEG	4367,00					1		1
Analítica	24,96	1	1	1	1	1	1	1
EKG	16,23			1	1			
Rx tórax	9,23			1	1			
Rx abdomen	9,23					1		1
TAC cráneo	55,38			1	1			
RMN cráneo	119,99			1	1			
TAC control	55,38			1	1			
RMN control	119,99			1	1			
Valoración anestesia	89,74			1	1			
PQ-Neurocirugía	23.132,00			1	1			
DBS 1º implante PC	24.758,00			1				
Recambio PC	16.078,00			1				
DBS 1º implante RC	33.110,00				1			
Recambio RC	24.430,00				1			
COSTES TOTALES		820,53	820,53	69.719,86	86.423,86	5397,80	555,07	5397,80

1€ = 1,0815 USD, 2025. LCIG, gel intestinal Levodopa/Carbidopa; FosLD/FosCD SC, infusión subcutánea de foslevodopa/foscarbidopa; LCEIG, gel intestinal Levodopa/Carbidopa/Entacapona; PEG, gastrostomía endoscópica percutánea; EKG, electrocardiograma; Rx, radiografía; TAC, tomografía computarizada; RMN, resonancia magnética; PQ-Neurocirugía, procedimiento quirúrgico Neurocirugía; DBS, Deep Brain Stimulation-Estimulación Cerebral Profunda; EP, enfermedad de Parkinson; RC, rechargable; PC, primary cell.

El estudio farmacoeconómico se realizó desde la perspectiva del Sistema Nacional de Salud (SNS) español y la tasa de descuento aplicada fue del 3,5% tanto en costes como en utilidades.

Los costes de los medicamentos analizados fueron a precio venta laboratorio (PVL), en € del año 2023, del nomenclátor español, calculándose el coste anual para las dosis medias estándar [21] (Tabla 2, Ref. [22, 23, 24, 25, 26, 27, 28, 29, 30, 31, 32, 33, 34]). Todos los costes se calcularon para 5 años para obtener las ratios de eficiencia.

**Tabla 2.** **Fármacos empleados en el tratamiento de la EP avanzada**.

	CN	Presentación	PVL	Principio activo	Dosis por unidad estándar	Coste	Dosis diaria mg [ref]	Unidades estándar diarias	Coste anual (€)
ApoGo® perfusión	74.401	5 jeringas	100,00€	apomorfina 5 mg/mL	50 mg apomorfina por jeringa	20	74,68 [34]	2	14.600
		10 mL							
Dacepton®	79.174	1 ampolla	40,00€	apomorfina 5 mg/mL	100 mg apomorfina por vial	40	74,68 [34]	0,75	10.950
perfusión		20 mL							
Duodopa® gel intestinal	66.547	7 cartuchos 100 mL	756,00€	1 mL contiene 20 mg de levodopa y 5 mg de carbidopa monohidrato.	2000 mg levodopa por cartucho	108	1300 ± 548 [27, 28, 29, 30]	1	39.420
Foslevodopa® sol. perfusión	88.677	7 viales	771,12€	1 mL contiene 240 mg de foslevodopa y 12 mg de foscarbidopa	2400 mg levodopa por vial	110,16	1621/1849 [29, 31, 32, 33]	1	40.208
		10 mL							
Lecigon®	86.092	7 cartuchos 47 mL	756,00€	1 mL contiene 20 mg de levodopa, 5 mg de carbidopa y 20 mg de entacapona	940 mg levodopa por cartucho	108	1164 [22, 23, 24, 25, 26]	1,5	59.130

CN, código nacional; PVL, precio venta laboratorio.

### 2.1 Medidas de Resultados: Eficacia y Utilidades

Las medidas de resultado utilizadas en este análisis se han tomado de estudios realizados en Europa [15, 35] en los que se realizó una modelización de Markov a partir de una revisión de ensayos clínicos pivotales y otros estudios clínicos para obtener la eficacia y utilidad de los distintos dispositivos, expresados en AVG y AVACs. Toda la información sobre los sujetos se encuentra disponible en un artículo publicado anteriormente [12].

El modelo de Markov desarrollado constaba de 12 estados de salud en EP avanzada y fueron una combinación de los estadios de H&Y, porcentaje de tiempo de vigilia en OFF, complicaciones (cirugía y gastrostomía endoscópica percutánea), eventos adversos (EA) con interrupción del tratamiento y posibilidad de cambio de tratamiento y muerte.

De la misma manera, se asumió que la mitad de los sujetos está en OFF (>14 h/día) cuando empieza el tratamiento y su estado puede variar [36, 37] en función de la enfermedad, tanto en cuanto gravedad, complicaciones incluso efectos adversos del tratamiento [36, 37, 38, 39, 40, 41].

Las efectividades (medidas en AVG) obtenidas con cada dispositivo fueron: 6385 para Apo-Go® y Dacepton®; 7055 para Lecigon® y Duodopa® gel; 8151 para Foslevodopa®, y 6495 para el dispositivo de deep brain stimulation-estimulación cerebral profunda (DBS) (Medtronic®).

Las utilidades obtenidas (medidas en AVAC) con cada dispositivo fueron: 2885 para Apo-Go® y Dacepton®; 3120 para Lecigon® y Duodopa® gel; 3605 para Foslevodopa®, y 2800 para el dispositivo de DBS (Medtronic®).

### 2.2 Análisis del Coste-Eficacia y Coste-Utilidad

Para comparar las distintas opciones terapéuticas se tuvieron en cuenta tanto la eficacia de cada opción (expresada en años de vida ganados) como las utilidades ganadas por cada opción terapéutica (expresadas en años vida ganados ajustados por calidad).

Para comparar entre dos opciones se realiza el calculó la ratio coste-eficacia/utilidad incremental, definida como el coste incremental (coste que hay que pagar de más) para ganar el beneficio obtenido de una opción respecto a otra. En nuestro caso:

Coste-eficacia/utilidad incremental:

ICER = (coste opción A – coste opción B) / (AVG opción A – AVG opción B)

ICUR = (coste opción A – coste opción B) / (AVAC opción A – AVAC opción B)

## 3. Resultados

El coste anual promedio de cada opción analizada (teniendo en cuenta las dosis diarias de los fármacos, los costes directos sanitarios y la tasa de descuento aplicada) fue: 13.421€ para Apo-Go®, 10.100€ para Dacepton®, 36.772€ para Duodopa® gel, 36.674€ para Foslevodopa®, 54.704€ para Lecigon® y finalmente 11.740€ y 14.553€ para Percept™ PC y RC respectivamente.

En la siguiente tabla (Tabla 3) se recogen los datos correspondientes al análisis de coste-eficacia y coste-utilidad con cada alternativa evaluada, a partir de la determinación de los costes directos sanitarios (Tabla 1) y la medicación empleada (Tabla 2) en relación a las medidas de eficacia (años de vida ganados por los pacientes) y utilidad (años de vida ganados ajustados por calidad).

**Tabla 3.** **Análisis farmacoeconómico de las alternativas disponibles en la EP avanzada**.

Dispositivos analizados	Nombre comercial	Ratio Coste (€)	
		ACE	ACU
		Eficacia (AVG)	Utilidad (AVAC)
Apomorfina	Apo-Go®	12.473	27.615
	Dacepton®	9387	20.782
Levodopa + Carbidopa + Entacapona	Lecigon®	46.047	104.000
Levodopa+Carbidopa	Duodopa® gel	30.953	69.909
Foslevodopa + Foscarbidopa	Foslevodopa®	26.808	60.319
Estimulación cerebral profunda (DBS)	Percept™ PC	10.732	24.874
	Percept™ RC	13.303	30.833

AVG, años de vida ganados; AVAC, años de vida ganados ajustados por calidad; ACE, Análisis de coste-eficacia; ACU, coste-utilidad

Como se observa, el coste para conseguir un beneficio de ganar 1 año de vida, osciló entre los 9387€ con Dacepton® y los 46.047€ con Lecigon®, la segunda alternativa más coste-eficaz fue la opción DBS - Percept® PC con un coste de 10.732€ por año de vida ganado.

Sin embargo, ganar 1 año de vida ajustado en perfecto estado de salud (AVAC), osciló entre los 20.782€ con Dacepton® y los 104.000€ con Lecigon®, la segunda alternativa más coste-eficaz fue la opción DBS - Percept® PC con un coste de 24.874€/AVAC.

**Fig. 1.**
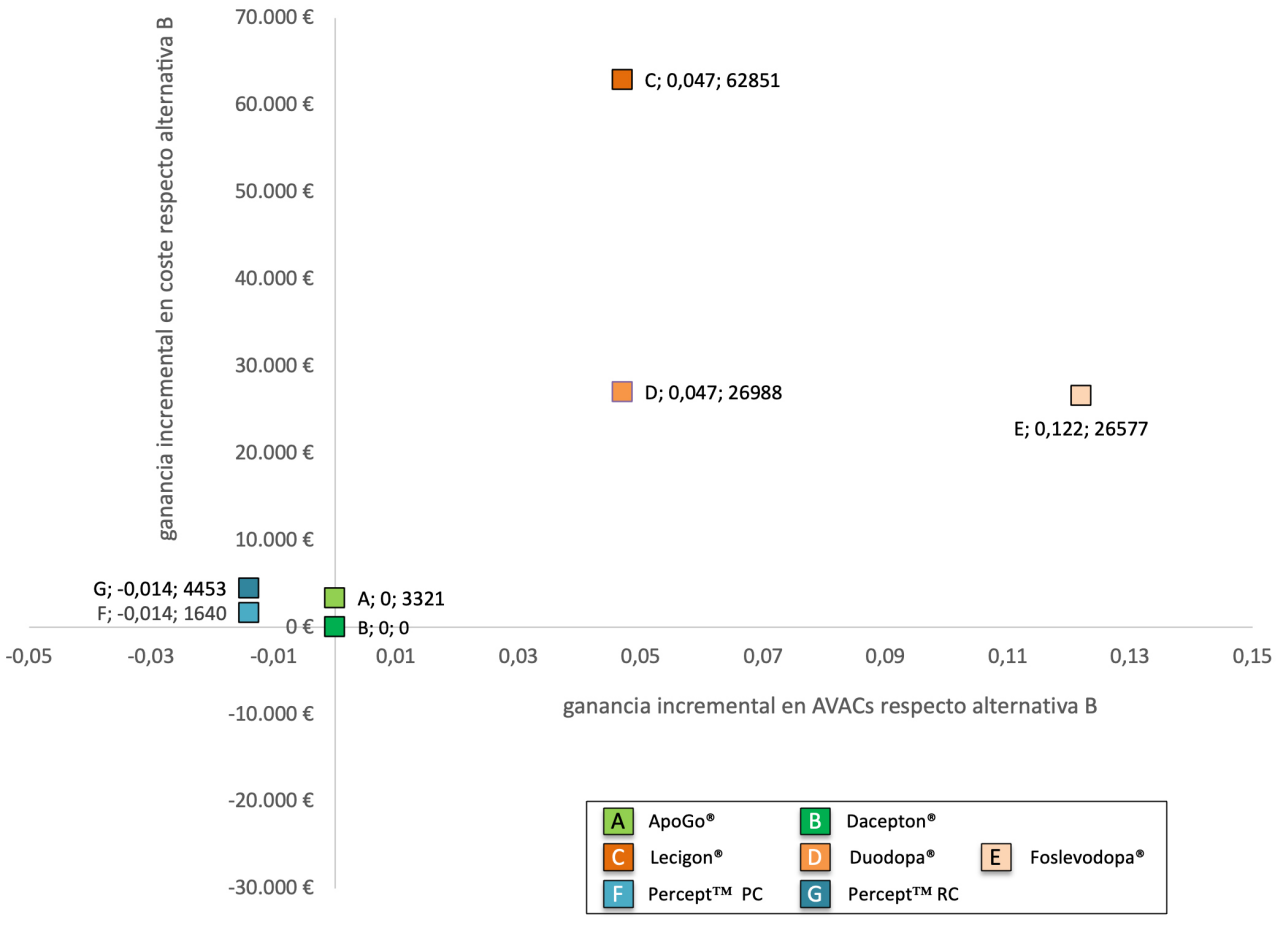
**Plano de eficiencia para el tratamiento de la EP avanzada (análisis coste-utilidad)**.

En la siguiente figura (Fig. 1) se muestra el denominado plano de coste-utilidad incremental, es decir cuanto más hay que pagar de una opción frente a otra y que beneficio se obtiene, todo expresado en términos de AVAC.

Al ser Dacepton® la opción más eficiente (menor ratio coste/utilidad), el análisis de coste-utilidad incremental se realizó comparándola frente a las otras alternativas, de manera que podamos conocer cuánto hay que pagar de más por conseguir 1 año más de vida ajustado por calidad.

Como se puede observar en la figura anterior (Fig. 1), hay tres opciones que son dominadas por Dacepton®: Apo-Go®, Percept™ PC y Percept™ RC, ya que tanto Percept™ PC como Percept™ RC ofrecen menos utilidad y mayor coste que Dacepton®; mientras que Apo-Go® ofrece la misma utilidad que Dacepton®, pero un mayor coste.

Por otro lado, para pasar de Dacepton® a Lecigon®, Duodopa® o Foslevodopa® aunque son más eficaces, también son más costosas, de manera que la ratio coste-utilidad incremental frente a Dacepton®, superan el denominado umbral de disponibilidad a pagar por ganar 1 AVAC (oscilando entre los 217.000 y más de 1.115.000 euros por AVAC ganado).

## 4. Discusión

El tratamiento y control de la EP supone un importante impacto económico para el SNS español, según datos publicados el coste puede alcanzar los 17.000€ anuales por pacientes [42]. Los costes totales aumentan con la progresión y la gravedad de la enfermedad, siendo los síntomas motores, el deterioro cognitivo y el dolor los principales predictores de costes. Las acciones terapéuticas dirigidas a ralentizar la progresión de la enfermedad y a controlar síntomas motores y cognitivos, así como el dolor, podrían ayudar a contener los gastos de la enfermedad [43].

El interés en la utilidad terapéutica y la eficiencia de los medicamentos en las decisiones de política sanitaria y farmacéutica está creciendo, especialmente con la evaluación de cómo contribuyen las innovaciones farmacéuticas al aumento marginal en la salud y su comparación con el uso de recursos (relación coste-eficacia/utilidad incremental).

### 4.1 Sobre los Dispositivos Analizados

Las terapias avanzadas ofrecen opciones efectivas para el tratamiento de la EP avanzada, pero es crucial seleccionar a los pacientes adecuados y considerar los posibles riesgos y costes asociados a cada terapia para garantizar resultados óptimos [12].

Las indicaciones para la DBS con EP avanzada incluyen fluctuaciones motoras y discinesias no controlables con medicamentos, una respuesta previa positiva al tratamiento con levodopa, una evolución entre 5 y 10 años, (más de 5 años desde el diagnóstico) y una edad inferior a 70 años según criterios core assessment program for surgical interventional therapies in Parkinson’s disease (CAPSIT-PD). Sin embargo, en la DBS no podrían participar los pacientes con síntomas extranigrales como deterioro cognitivo, disartria/disfagia, bloqueos motores resistentes a levodopa, además, la presencia de demencia es una contraindicación, al igual que los episodios psicóticos o depresivos graves.

En cuanto a Duodopa® (gel intestinal Levodopa/Carbidopa (LCIG)) y Lecigon® (gel intestinal Levodopa/Carbidopa/Entacapona (LCEIG)), se recomiendan para pacientes sin deterioro cognitivo significativo y que tienen una respuesta a levodopa con buen estado ON. Además, las contraindicaciones de LCIG y LCEIG incluyen la presencia de sintomatología axial en forma de alteración del equilibrio, disfagia y congelación de la marcha, así como la existencia de una cirugía abdominal previa, que dificultaría la realización de una gastroyeyunostomía.

Mientras que la infusión continua de apomorfina (CSAI: Dacepton®, Apo-Go®) es una opción para aquellos con problemas en el manejo de las fluctuaciones OFF y discinesias, a pesar de una terapia oral optimizada. CSAI puede no ser adecuada para aquellos con depresión respiratoria y/o insuficiencia hepática, demencia grave o enfermedad psicótica. La falta de adherencia al tratamiento debido a la reversibilidad inmediata del mismo y la posibilidad de aparición de nódulos (tratables con cambios en la zona de punción o con ultrasonidos cuando ya se han producido) son también aspectos a tener en cuenta.

Las complicaciones de la infusión subcutánea de Foslevodopa/Foscarbidopa (Foslevodopa®) en pacientes con EP avanzada pueden incluir celulitis, nódulos, infecciones en el lugar de perfusión y en el tracto urinario, que suelen ser las complicaciones más frecuentes, y complicaciones derivadas del sistema de infusión, como infecciones o daños en el dispositivo de infusión.

### 4.2 Respecto a la Eficiencia (Ratio Coste-Utilidad)

En nuestro estudio, es evidente que los costes por paciente tratado fueron muy superiores debido al estado avanzado en los pacientes. Al igual que en otros estudios [17, 44] en los que los pacientes con EP avanzada tenían una carga significativamente más alta sobre los recursos de atención de la salud en comparación con los pacientes bien controlados con tratamiento oral o que sólo requerían ajustes de la medicación oral.

En un estudio realizado en nuestro país mediante análisis de datos en vida real, los costes medios anuales por paciente respecto a DBS y Duodopa® son semejantes a los obtenidos en nuestra publicación, no así con CSAI, donde hubo notables diferencias [45]. Estas diferencias de resultados entre diversos estudios pueden deberse a factores contextuales específicos y a las características de cada sistema sanitario. Los costes aumentan en fases avanzadas de la enfermedad, y la refractariedad provoca una menor eficacia en el control de los síntomas y, en muchos casos, el abandono o la interrupción del tratamiento [46, 47].

El conocer la ratio coste-eficacia o coste-utilidad de un tratamiento implica conocer también el máximo que el sistema sanitario está dispuesto a pagar por ganar un AVAC, esto se denomina disponibilidad a pagar (DAP) o coste añadido dispuesto a pagar. Aunque en nuestro país no hay un umbral especificado por las autoridades sanitarias, las ratios de coste/utilidad estándar oscilan entre 22.000 y 25.000 € por AVAC [48] o incluso superior en situaciones especiales (hasta 60.000€/AVAC) [49]. En nuestro estudio la única opción que superó estos umbrales fue Lecigon®.

Los datos muestran que la opción más eficiente fue la Dacepton®, seguida de DBS (Percept™ PC) y de Apo-go®. Pasar de la opción de SCAI a cualquiera de las otras alternativas no resultoéficiente (siendo en ambos casos el coste-utilidad superior a 30.000€/AVAC). Existe una presentación de Apo-go® no comercializada hasta el momento de este análisis, que igualaría en costes a Dacepton® siempre que las dosis superasen los 0,50 mg/día.

Los resultados de diferentes análisis farmacoeconómicos ponen de manifiesto que las ratio coste-utilidad son sensibles a los cambios en los diferentes parámetros analizados: costes de los recursos empleados, eficacia a largo plazo de las opciones comparadas, y proporción de pacientes que necesitan más recambios en los dispositivos [36, 45, 46, 47, 50].

El tratamiento con estos dispositivos se asocia a una reducción del tiempo en OFF y con una ganancia sustancial de AVAC, lo que permite a los pacientes seguir siendo independientes durante más tiempo y, por tanto, reducir la carga social y de cuidados informales.

## 5. Conclusiones

Estos resultados permiten obtener información adicional sobre la eficiencia de los tratamientos que deben servir en la toma de decisiones en el manejo de la enfermedad de Parkinson avanzada, y de este modo conseguir una mejor gestión de los recursos.

## Data Availability

Los conjuntos de datos utilizados y analizados durante el presente estudio están disponibles a pedido razonable al autor de correspondencia.

## Contribuciones de los Autores

NGS, LGT, and AJGR han hecho contribuciones sustanciales a la concepción y diseño y han participado en la redacción del manuscrito. NGS y AJGR adquisición de datos, han desarrollado la adquisición de los datos, el análisis estadístico y la interpretación de los datos. Todos leyeron y aprobaron el manuscrito final. Todos los autores participaron lo suficiente en el trabajo y aceptaron ser responsables de todos sus aspectos.

## Aprobación Ética y Consentimiento Informado

No aplicable.

## Agradecimientos

No aplicable.

## Financiación

Esta investigación no recibió financiación externa.
